# Very low enalapril and enalaprilat exposure *via* human milk: a case report from the ConcePTION project

**DOI:** 10.3389/fphar.2025.1727499

**Published:** 2026-01-20

**Authors:** Emily Jacobs, Michael Ceulemans, Nina Nauwelaerts, Siemon de Nys, Kathleen J. Claes, Pieter Annaert, Kristel Van Calsteren, Anne Smits, Karel Allegaert, Martje Van Neste

**Affiliations:** 1 Faculty of Medicine, KU Leuven, Leuven, Belgium; 2 Clinical Pharmacology and Pharmacotherapy, Department of Pharmaceutical and Pharmacological Sciences, KU Leuven, Leuven, Belgium; 3 L-CY, KU Leuven Child & Youth Institute, Leuven, Belgium; 4 Research Foundation Flanders (FWO), Brussels, Belgium; 5 Drug Delivery and Disposition, Department of Pharmaceutical and Pharmacological Sciences, KU Leuven, Leuven, Belgium; 6 ESQlabs GmbH, Saterland, Germany; 7 BioNotus CommV, Niel, Belgium; 8 Department of Nephrology and Renal Transplantation, University Hospitals Leuven, Leuven, Belgium; 9 Department of Microbiology, Immunology and Transplantation, Nephrology and Renal Transplantation Research Group, KU Leuven, Leuven, Belgium; 10 Department of Development and Regeneration, KU Leuven, Leuven, Belgium; 11 Department of Obstetrics and Gynaecology, Division of Foetomaternal Medicine, University Hospitals Leuven, Leuven, Belgium; 12 Neonatal Intensive Care Unit, University Hospitals Leuven, Leuven, Belgium; 13 Department of Hospital Pharmacy, Erasmus University Medical Center, Rotterdam, Netherlands

**Keywords:** breastfeeding, case report, enalapril, enalaprilat, human milk, lactation, nephropathy, pharmacokinetics

## Abstract

**Introduction:**

Ongoing maternal and clinical hesitancy in breastfeeding-related shared decision-making is driven by limited safety data on maternal pharmacotherapy. Theoretical exposure to maternal enalapril and its active metabolite enalaprilat in breastfed infants has previously been reported in two studies of eight mother-infant pairs. However, actual infant plasma concentrations remain uncharacterized.

**Methods:**

A 30-year-old white woman started enalapril (5 mg, 1x/day) for IgA nephropathy at 11 weeks postpartum while exclusively breastfeeding. On day 101 postpartum, 25 days after therapy start, she collected six steady-state milk samples over 24 h, along with two maternal and one infant blood sample, used to calculate milk-to-plasma (M/P) ratio and estimated infant exposure. Samples were analyzed using liquid chromatography with tandem mass spectrometry. Maternal and infant health information was concurrently collected *via* structured questionnaires.

**Results:**

Low levels of enalapril (0.01–1.22 ng/mL) and enalaprilat (0.32–0.77 ng/mL) were measured in human milk. Using 200 and 150 mL/kg/day milk intake, estimated daily infant dosage was 78.32 ng/kg/day and 58.82 ng/kg for enalapril and 124.45 ng/kg/day and 93.34 ng/kg/day for enalaprilat, corresponding to a relative infant dose (RID) of 0.097% and 0.073% for enalapril. Infant enalapril and enalaprilat plasma concentrations were below the lower limit of quantification.

**Discussion:**

These data support evidence of minimal transfer of enalapril and enalaprilat into human milk, suggesting low risk to breastfed infants, and may help address uncertainties in current clinical guidelines. This case report provides detailed maternal pharmacokinetic data for both compounds in human milk, alongside estimated infant exposure at 3 months postpartum.

## 1 Introduction

Enalapril (molecular weight: 376.45 g/mol) is an orally active angiotensin-converting enzyme (ACE) inhibitor, widely prescribed for chronic cardiorenal conditions in adults, including hypertension, heart failure, and off-label, chronic kidney disease (CKD) (Faruqi et al., 2025). In its prodrug form, enalapril is only transiently detectable in plasma, with peak concentrations occurring within 1 hour of oral administration. Subsequently, approximately 60% is rapidly hydrolyzed by hepatic carboxylesterases to enalaprilat (Davies et al., 1984; Ulm et al., 1982). Enalapril exhibits an oral bioavailability of approximately 40%, and its antihypertensive and antiproteinuric effects are mediated by its active metabolite, enalaprilat (Kubo and Cody, 1985; Ulm et al., 1982). Oral administration of enalaprilat itself results in negligible systemic absorption, with a bioavailability of approximately 3% (Ulm, 1983)), which accounts for the clinical use of enalapril rather than enalaprilat, and is also relevant when considering human milk related exposure. Furthermore, enalaprilat has a substantially longer therapeutic half-life than enalapril, of approximately 11 h in adults (Marte et al., 2023), with peak plasma concentrations occurring 3.5–4.5 h post-administration (Ulm et al., 1982). Elimination occurs primarily *via* the kidneys, with 61% of the dose recovered in urine and 33% in feces (Ulm et al., 1982).

Enalapril is contraindicated during pregnancy because of its teratogenic effects (Bullo et al., 2012) and therefore, early introduction of ACE therapy following delivery is often critical to initiate or restore disease control in women with CKD or postpartum hypertension (McCormack and Brennand, 2024). In the context of lactation, pharmacokinetic and safety data of enalapril and enalaprilat remain limited and somewhat outdated (Huttunen et al., 1989; Redman et al., 1990), which creates considerable challenges for shared decision-making (Tabacova and Kimmel, 2001). Nevertheless, enalapril remains the preferred ACE inhibitor in breastfeeding women given current evidence (Beardmore et al., 2002; Piotrkowicz et al., 2025). Furthermore, its use has long been restricted in children under 20 kg in European countries due to insufficient safety data in this population, exacerbating fears of infant exposure (Hurtado and Moffett, 2007; Van Hecken et al., 2020). Recently, however, the clinical pharmacokinetics were summarized by the Labeling of Enalapril from Neonates up to Adolescents (LENA) consortium (Faisal et al., 2021), expanding therapeutic options for pediatric heart failure (EMA, 2023).

Breastfeeding confers significant long-term health benefits for both mother and child (Chowdhury et al., 2015; Davis et al., 2022; Duijts et al., 2010; Sivasankar et al., 2025) and is widely regarded as the ideal form of infant nutrition (Andreas et al., 2015; Tain et al., 2025). Current recommendations support exclusive breastfeeding for the first 6 months, with continuation alongside gradual introduction of complementary foods for 2 years, or longer, depending on infant and maternal preference (World Health Organization, 2023). Nevertheless, global breastfeeding rates remain suboptimal: in the United States for instance, despite 83.2% initiation rates, only 35.9% of infants are breastfed at 1 year (CDC, 2022). In mothers with chronic disease outcomes are even less favorable. Affecting 10%–30% of the breastfeeding population, chronic diseases are consistently associated with lower rates of sustained exclusive breastfeeding, with this demographic up to 2.5 time more likely to cease breastfeeding prematurely than their healthy peers (Jølving et al., 2016; Pilgrim et al., 2025; Scime et al., 2022).

Among the myriad factors contributing to these suboptimal outcomes, polypharmacy, and specifically, concerns over milk-mediated drug exposure in infants appear salient (Parnizari et al., 2025; Sachs et al., 2013). In the absence of adequate safety data on human milk transmission and inconsistent regulatory guidance (ACOG, 2019; Colaceci et al., 2015; EMA, 2025; EMA, 2023; FDA, 1985), clinical decisions are often made with considerable uncertainty. Particularly as only 16% of European Medicines Agency (EMA) and US Food and Drug Administration (FDA) lactation labelling include human data (Kappel et al., 2023). Faced with the need to balance the well-established benefits of breastfeeding against optimal disease management, in a context shaped more by assumed than demonstrated risks, breastfeeding hesitancy or alternatively, maternal non-adherence and off-label use, becomes the natural outcome (Colaceci et al., 2015).

Here, we present a case study from the UmbrelLACT study and the Innovative Medicine Initiative (IMI) ConcePTION project^1^, reporting both the concentrations of enalapril and its active metabolite enalaprilat in human milk, as well as the actual and estimated infant exposure through human milk. The report was written in accordance with the CARE guidelines and the ‘Guidelines for reporting cases of medication use during lactation’ (Anderson, 2022; Gagnier et al., 2013).

## 2 Methods

The current research report describes a mother-infant pair enrolled in the UmbrelLACT study (NCT06042803), whose protocol and ethics approval from the Ethics Committee Research UZ/KU Leuven have been previously reported (Hansson et al., 2025; Van Neste et al., 2024). Prior to enrolment, the mother provided written informed consent forms for herself and her infant. After inclusion, she completed multiple self-report structured questionnaires, covering maternal and infant clinical data, such as biometrics, comorbidities, intake of medication in the 3 days preceding sample collection and general information concerning pregnancy and breastfeeding (Van Neste et al., 2024).

It is important to note that the mother’s decision to take enalapril while breastfeeding, as well as any aspect of her clinical care, were independent of the study team.

### 2.1 Sample collection

Starting from enalapril intake, six human milk samples were collected intermittently at steady state over a period of 24 h. During this time, the infant was not put to the breast; however, feeding practices were otherwise left to the discretion of the mother. Milk was expressed at home for approximately 20 min using an electric pump, respecting the usual feeding schedule of the mother-infant pair, in accordance with UmbrelLACT protocol. The total volume obtained from both breasts at each feeding session was collected, with foremilk and hindmilk combined, and the total volume was recorded. Subsequently, a 10 mL sample was retained from each timepoint for bioanalysis of enalapril and enalaprilat concentrations. For further details regarding the milk collection methodology, please refer to the previously published protocol (Van Neste et al., 2024).

Maternal blood samples were collected within 1 h of the first and last milk expression sessions in ethylenediaminetetraacetic acid (EDTA) tubes, and an infant plasma sample was obtained close to the first breastfeeding session of the study period.

All samples were stored in the mother’s refrigerator for up to 24 h, at which time they were transported for long-term storage at −80 °C on ice. Concurrent with sample collection, the mother also completed standardized questionnaires to collect maternal biometrics and clinical data, together with general health outcomes of the infant (Van Neste et al., 2024).

### 2.2 Bioanalytical method for enalapril and enalaprilat

The samples were analyzed using liquid chromatography with tandem mass spectrometry (LC-MS/MS), see Supplementary Material. All data analyses were conducted in Microsoft Excel v.2508, Microsoft 365. The calculations used to estimate infant exposure are detailed below.

### 2.3 Pharmacokinetic calculations

Pharmacokinetic parameters and exposure metrics were calculated using the following equations.

Equation 1 Milk-to-Plasma Ratio.

The milk-to-plasma ratio was calculated to estimate the extent of drug transfer from maternal plasma into human milk.

$$\frac{M}{P}ratio=\frac{AUC_{human\;milk}}{AUC_{plasma}}$$

Equation 2 *Relative Infant Exposure*.

Relative infant exposure was calculated to quantify the proportion of the maternal dose ingested by the breastfed infant.

$$Relative\;infant\;exposure\;\left(\%\right)=\frac{Infant\;plasma\;concentration\;\left(\frac{ng}{mL}\right)}{Maternal\;plasma\;concentration\;\left(\frac{ng}{mL}\right)}*100$$

Equation 3 *Daily Infant Dose as a function of patient data for the exclusively breastfed infant*.

The daily infant dose was calculated using patient-specific data for an exclusively breastfed infant to estimate actual drug intake.

$$DID\;\left(\frac{\frac{ng}{kg}}{day}\right)=\sum_{i=1}^{n}\left(\frac{Milk\;Concentration_{i}\left(\frac{ng}{L}\right)*Milk\;Volume_{i}\left(L\right)}{Infant\;weight\;\left(kg\right)}\right)*100$$

Equation 4 *Daily Infant Dose as a function of standardized milk intake*.

The daily infant dose was also estimated using a standardized milk intake to allow comparison across studies.

$$DID\;\left(\frac{\frac{ng}{kg}}{day}\right)=\text{Average Steady State Milk Concentration }\left(\frac{ng}{L}\right)*Infant\;Milk\;Intake\;\left(\frac{\frac{L}{kg}}{day}\right)$$

Equation 5 *Relative Infant Dose*.

The relative infant dose was calculated as the infant dose normalized to the maternal weight-adjusted dose to assess breastfeeding safety.

$$RID\;\left(\%\right)=\frac{DID\;\left(\frac{\frac{ng}{kg}}{day}\right)}{Daily\;Maternal\;Dose\;\left(\frac{ng}{day}\right)/\;Maternal\;Weight\;\left(kg\right)}*100$$

Equation 6 *Relative Infant Therapeutic Dose*.

The relative infant therapeutic dose was calculated by comparing the estimated infant exposure to the established therapeutic dose in infants.

$$RID_{therapeutic}\left(\%\right)=\frac{DID\;\left(\frac{\frac{ng}{kg}}{day}\right)}{Daily\;Therapeutic\;Infant\;Dosage\;\left(\frac{\frac{ng}{kg}}{day}\right)}*100$$

## 3 Results

### 3.1 Case description: patient information and therapeutic interventions

The patient was a 30-year-old, primigravida, white woman, diagnosed with biopsy-confirmed IgA nephropathy during pregnancy, and treatment with 5 mg Enalapril, E.G., (Eurogenerics NV, Brussels, Belgium), a generic formulation of the ACE inhibitor enalapril was initiated in postpartum at 11 weeks (day 76) postpartum after the patient presented with proteinuria. At the time of sample collection, she was 14 weeks postpartum (day 101), weighed 61.8kg, and measured 168cm, corresponding to a BMI of 21.9 kg/m^2^. She reported following a normal diet and no recreational drug, alcohol or cigarette use. No comorbidities or chronic diseases were reported, aside from her kidney condition, and she reported not taking any other medications or supplements in the weeks preceding sample collection. At time of sampling, the patient’s serum creatinine was 1.26 mg/dL and her eGFR was 57 mL/min/1.73 m^2^ corresponding to stage 3 chronic kidney disease (CKDG3). The dose of enalapril was not further increased because of low blood pressure.

The infant was a healthy, term male (38 weeks gestation), weighing 3.04 kg and measuring 48 cm at birth. At the time of sample collection (aged 101 days), the infant was exclusively breastfed, thus was exposed to maternal pharmacotherapy, and weighed 5.84 kg and measured 59 cm. The infant received daily vitamin D supplementation (D-Cure 2400 IU/mL, SMB-GALEPHAR, Marche-en-Famenne, Belgium), in line with the current recommendations (Van de Walle et al., 2024).

### 3.2 Timeline

**FIGURE 1:**
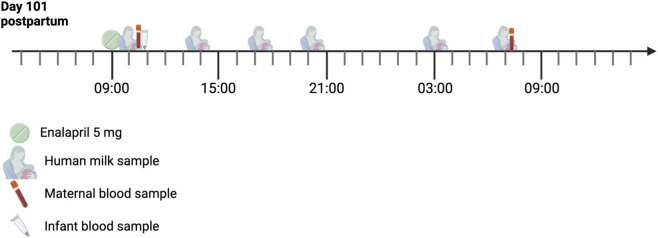
Timeline of the 24-h sampling day. The mother took 5 mg enalapril once daily, and all samples were obtained within 24 h.

The timeline of the 24-h sampling day is illustrated in Figure 1. The first milk sample was collected 1 hour after enalapril intake, with both maternal and infant plasma samples within 30 min of milk sample collection.

### 3.3 Follow-up and outcomes

#### 3.3.1 Maternal and infant health

Maternal clinical data and medication use, as well as infant data, including clinical information, growth, hospitalizations, adverse effects, and medication use, were collected *via* a standardized self-report questionnaire completed by the mother. No adverse events or hospital admissions were reported for the infant during the 2 months following sample collection (21 weeks postpartum). Continued daily vitamin D supplementation was confirmed for the infant.

#### 3.3.2 Collection of maternal samples

Six human milk samples were collected over the 24-h period at steady-state, as the medicine had been taken consistently prior to sampling, 5 mg once every 24 h, according to the mother’s report. Two maternal blood samples were collected within 30 min of the first and final milk expression sessions, with the first sample taken within 2 h of the enalapril intake (Figure 1). The total milk volume expressed per session ranged from 100–190 mL, with an average of 135 mL. A summary of the maternal sample characteristics is provided in Table 1. In these maternal milk samples, two enalapril concentrations were below the lower limit of quantification (LLOQ; 0.2 ng/mL) and were estimated, albeit with greater uncertainty.

**TABLE 1:** Summary of the maternal samples collected and the concentrations of enalapril and enalaprilat over 24 h.

Days postpartum	Time of sampling (hh:mm)	Time since enalapril intake (hours)	Sample type	Concentration (ng/mL)	
				Enalapril	Enalaprilat
101	10:06	1.10	Milk	0.70	0.72
	10:29	1.48	Plasma	51.74	32.74
	13:52	4.87	Milk	1.22	0.77
	17:17	8.28	Milk	0.41	0.70
	20:13	11.21	Milk	0.22	0.77
102	03:03	18.05	Milk	0.01^a^	0.43
	07:02	22.03	Milk	0.04^a^	0.32
	07:27	22.45	Plasma	0.24	5.93

^a^ Measurements below the lower limit of quantification were estimated and likely carry a greater margin of error.

#### 3.3.3 Human milk and maternal plasma pharmacokinetics

**FIGURE 2:**
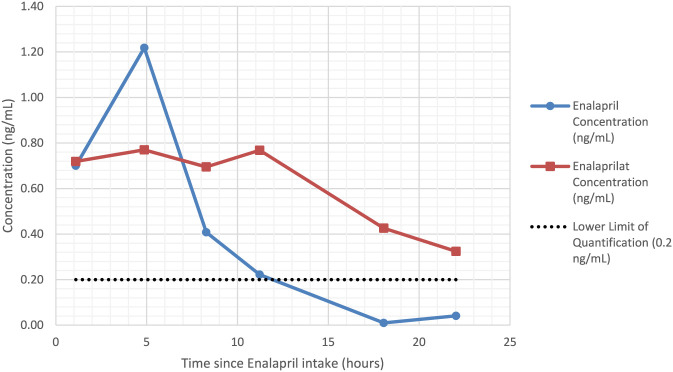
Steady-state human milk concentration-time profiles of enalapril and enalaprilat at 13 weeks postpartum.

The concentration time profiles of enalapril and enalaprilat in the six human milk samples are shown in Figure 2. Enalapril concentrations in human milk samples ranged from below the LLOQ (estimated at 0.01 ng/mL) to 1.22 ng/mL, while enalaprilat concentrations ranged from 0.32 to 0.72 ng/mL. In maternal plasma, concentrations of enalapril and enalaprilat ranged from 0.24–51.74 ng/mL and 5.93–32.74 ng/mL, respectively. Calculated using the trapezoidal rule, the mean AUC_24h_ in human milk was 8.21 ng*h/mL for enalapril and 13.03 ng*h/mL for enalaprilat, with mean average steady-state concentration (C_av,ss_) values of 0.39 ng/mL and 0.62 ng/mL, respectively.

#### 3.3.4 Milk-to-plasma (M/P) ratio

The plasma AUC_24h_ could not be calculated for the patient, as only two plasma samples were obtained, preventing accurate calculation. Therefore, the mean plasma ${\text{AUC}}_{0-\infty }$ of both enalapril and enalaprilat were taken from the literature (137 ng*h/mL and 265 ng*h/mL respectively), as AUC_24h_ could not be extracted from this publication (Schwartz et al., 1985). To our knowledge however, this is the only study reporting the pharmacokinetics of a single daily 5 mg dose of enalapril, albeit in non-pregnant, non-postpartum adults, with unknown kidney function.

The patient’s milk AUC_24h_ was therefore combined with the plasma ${\text{AUC}}_{0-\infty }$ from Schwartz et al. (1985) to calculate the milk-to-plasma (M/P) ratio (see Equation 1). The M/P ratios were 0.060 and 0.049 for enalapril and enalaprilat, respectively.

Single time point M/P ratios were also calculated using the coincident milk and plasma samples (M1/P1 within ∼30 post-dose and M6/P2 ∼22 h post dose), yielding a mean M/P ratio of 0.093 for enalapril (0.21 LLOQ/2) and 0.038 for enalaprilat.

#### 3.3.5 Infant plasma sample and drug exposure

A single plasma sample was collected from the breastfed infant, in which both enalapril and enalaprilat were below the LLOQ (0.2 ng/mL). Because enalapril and enalaprilat concentrations in infant plasma samples were below the LLOQ, pharmacokinetic calculations were estimated using the LLOQ value to derive a conservative upper-bound estimate. Calculated according to Equation 2, the relative infant exposure was found to be ≤0.0039% for enalapril and ≤0.0061% for enalaprilat.

As DID can fluctuate considerably depending on an infant’s milk intake, exposure was also calculated using the standardized milk intakes of 200 mL/kg/day (early infancy intake) and 150 mL/kg/day (standard infancy intake) to enhance generalizability for exclusively breastfed infants (Cloostermans et al., 2024) (Equations 4 and 5). The relative infant dose (RID), which relates the infant-specific DID (Equation 3) *via* human milk to the maternal daily dose corrected for weight (Equation 5), was 0.086% for enalapril. Accounting for molar exposure to both enalapril and enalaprilat respectively, the combined RID was 0.202%. When adjusted for standardized milk intake (DID derived from Equation 4), the RID for enalapril was 0.097% and 0.073% for 200 mL/kg/day and 150 mL/kg/day respectively. Total RID accounting for molar exposure to both prodrug and metabolite was then 0.261% and 0.196% for 200 mL/kg/day and 150 mL/kg/day respectively.

## 4 Discussion

This paper reports the concentrations of enalapril and enalaprilat in human milk, the maternal M/P ratio and the infant exposures, assessed by an infant blood sample, as well as the health outcomes of the breastfed infant at 13 and 21 weeks postpartum.

Our findings show that both enalapril and enalaprilat were detectable in human milk at very low levels, corresponding to an RID of <1%, well below the generally accepted threshold of 10% (Anderson, 2018). The low transfer to the infant was further supported by infant plasma concentrations of both enalapril and enalaprilat below the LLOQ, as well as the absence of reported adverse events during follow-up. It is worth noting, however, that infant blood pressure, which may act as an indicator of adverse events, was not measured (Kiesel et al., 2023). Furthermore, it must be acknowledged that, aside from hospitalizations, unspecified health problems and prescribed medication, no additional adverse events were explicitly monitored in the 2 months following sampling. When comparing the daily infant dose to the therapeutic pediatric dose of enalapril (0.1 mg/kg), the exposure *via* milk represented less than 0.08%, reinforcing the minimal risk of clinically-relevant drug exposure, particularly in the face of the well-established benefits of breastfeeding. These data add to the limited existing literature on the pharmacokinetics and safety of enalapril during lactation, which to date includes only eight historically reported mother-infant pairs (Huttunen et al., 1989; Redman et al., 1990), and provide further support for the safety of enalapril during lactation. Moreover, this paper is the first of its kind to measure infant plasma concentrations of enalapril and enalaprilat in a breastfed infant whose mother was receiving 5 mg of enalapril daily, thereby providing direct evidence of minimal exposure through human milk during pharmacotherapy.

Notably, our quantitative results align with previous case series of enalapril during breastfeeding, where milk concentrations were low (M/P ratios ≤0.043 and 0.31) and infant outcomes were reassuring (Huttunen et al., 1989; Redman et al., 1990). Interestingly, although the studies suggested a dose-dependent relationship between enalapril dose and enalaprilat detection in human milk, with Huttunen et al. unable to detect enalaprilat 4 hours after a 5–10 mg dose, we detected enalaprilat in human milk up to 22 h post-administration, albeit at low concentrations. This prolonged presence may be explained by the impaired renal function in our patient, which is associated with higher plasma drug concentrations (Elung-Jensen et al., 2003; Kelly et al., 1986). Among the mothers in previous studies, renal function was reported for only one participant with chronic glomerulonephritis (serum creatinine: 0.89 mg/dL, creatinine clearance: 60 mL/min/1.73 m^2^). Our findings are therefore particularly reassuring, even in the context of moderate renal impairment (CKDG3), since infant exposure remained well below clinically relevant thresholds. Furthermore, given the enhanced sensitivity of contemporary bioanalysis techniques, our results are even more compelling.

The limited transfer of enalapril and enalaprilat to human milk, even in the context of CKDG3, can be explained by their pharmacological properties. Both enalapril and enalaprilat are weak acids with low lipophilicity, low-to-moderate molecular weights (enalapril 376.45 and enalaprilat 384.43), and moderate protein binding, up to 50% (Davies et al., 1984; European Bioinformatics Institute, 1985; Ranadive et al., 1992). While their small size and moderate protein binding could favor passive diffusion, the combination of weak acidity and low lipophilicity likely limits transfer into slightly acidic human milk (Anderson, 2018; Beardmore et al., 2002). Consequently, ACE inhibitors, and therefore enalapril, present some of the safest medications to use during breastfeeding in the management of hypertension (Piotrkowicz et al., 2025). Although enalapril currently remains the preferred option, captopril use during breastfeeding is also accepted (Devlin and Fleiss, 1981; Ghelfi et al., 2021; Huttunen et al., 1989; Parnizari et al., 2025; Piotrkowicz et al., 2025; Redman et al., 1990). Exploratory findings further support the safe use of quinapril, benazepril, perindopril and lisinopril, which appear to demonstrate RIDs of 1.6%, <0.14%, 0.0005%–0.2% and 0.06%, respectively, with no documented infant adverse effects (Begg et al., 2001; Chugh et al., 2025; Kaiser et al., 1989; Leggett et al., 2020).

Despite these reassuring findings, current clinical guidelines remain inconsistent, contributing to ongoing uncertainty among clinicians and mothers (Parnizari et al., 2025). While the American College of Obstetricians and Gynecologists (ACOG, 2019) considers low-dose use compatible with breastfeeding, the FDA, as well as the EMA adopt a more cautious stance, advising mothers to choose between treatment and breastfeeding, particularly in preterm infants and neonates (Colaceci et al., 2015; EMA, 2025; FDA, n.d.; MHRA, 2009). This hesitance is likely maintained by the understanding of ontogenetic changes in enalapril metabolism and increased ACE-sensitivity of neonatal kidneys (Boberg et al., 2017; Kearns et al., 2003; Ku et al., 2017). Furthermore, case reports have been published, albeit infrequently, detailing enalapril-induced adverse renal events including hypotension, renal failure and acute kidney injury in neonates who had received therapeutic doses of enalapril (0.07–0.1 mg/kg) for hypertension (Kanic et al., 2021; Schilder and Van den Anker, 1995). It is, however, worth noting that no adverse events have been reported in infants following exposure to enalapril through human milk (Piotrkowicz et al., 2025) and that the European project LENA is expected to address the knowledge gap surrounding pediatric use of the drug in the context of pediatric heart disease (Bajcetic et al., 2019).

Several methodological limitations should be acknowledged. First, milk sampling and preliminary storage were completed at the patient’s home, which may introduce uncertainty regarding strict protocol adherence and storage conditions, but also reflects the pragmatism and feasibility of the UmbrelLACT study (Van Neste et al., 2024). Second, milk volume measurements using an electric pump may be inaccurate. Yield could be overestimated because infants do not routinely empty both breasts (Anderson and Sauberan, 2016), or underestimated in some mothers due to suboptimal stimulation or stress associated with pumping (Kent et al., 2003). Although the risk of intraindividual variability remains low (Gardner et al., 2015), variability in hind- and foremilk fat content could influence measured drug concentrations. While enalapril is not strongly lipophilic, differences in milk composition may still affect sample concentrations and remain unknown for enalapril. Third, insufficient maternal plasma samples were available to calculate M/P ratios over the full 24 h, necessitating reliance on literature-derived plasma AUC from non-breastfeeding, non-postpartum adults, which do not account for the physiological changes of pregnancy or lactation (Van Neste et al., 2023). Furthermore, these reference AUC values were obtained from adults without CKD and represented ${\text{AUC}}_{0-\infty }$ rather than 24-h values, likely increasing the error margins in M/P ratios and overall infant exposure estimates, leading to an overall underestimation, although this would not impact calculations of DID and RID. Finally, the infant included in this report was older than 3 months, limiting generalizability of our findings to neonates in the first weeks of life and to prematurely born infants, who may be more vulnerable. Nevertheless, the low measured infant plasma concentration provides direct evidence that systemic exposure was minimal.

Despite these limitations, our findings add to the reassuring evidence base for enalapril during lactation in patients with mildly impaired renal function. Infant exposure appears negligible, with no short-term adverse outcomes reported. Given the substantial benefits of breastfeeding for both mother and infant, these results suggest that maternal enalapril therapy may be compatible with breastfeeding in similar clinical scenarios. To strengthen the evidence base, further systematic pharmacokinetic studies are needed, particularly in the early postpartum period and among high-risk populations, following structured frameworks such as the Milk4Baby decision tree to guide the selection of appropriate methodologies (Monfort et al., 2025). In addition, physiologically based pharmacokinetic (PBPK) modeling present a way to predict infant exposure using minimal clinical samples, with the ability to account for changes in maternal physiology and infant enzyme maturation (Nauwelaerts et al., 2023). This approach, evaluated by clinical real-world data, could be used to refine clinical recommendations and reduce unnecessary treatment discontinuation or avoidance of breastfeeding, in addition to contributing to the establishment of much needed teratology and breastfeeding-related information services (Ceulemans et al., 2022).

In conclusion, this case report is the first to include infant plasma sampling in the investigation of mother-infant enalapril and enalaprilat transfer. Overall, we found low concentrations of enalapril and enalaprilat in human milk, resulting in negligible infant exposure, with no short-term adverse events observed. Taken with prior reports, these findings support the safety of enalapril therapy during breastfeeding. However, additional data collection and PBPK simulations, are needed to capture pharmacokinetic variability, particularly in neonates and preterm infants, and to confirm safety across different maternal and infant populations.

## Data Availability

The datasets presented in this article are not readily available because of ethical and privacy restrictions. Requests to access the datasets should be directed to the corresponding author.

## Footnotes

1
https://www.imi-conception.eu/

## References

[B1] ACOG (2019). ACOG practice bulletin no. 203: chronic hypertension in pregnancy. Obstet. Gynecol. 133, e26–e50. 10.1097/AOG.0000000000003020 30575676

[B2] Anderson P. O. (2018). Drugs in lactation. Pharm. Res. 35, 1–13. 10.1007/s11095-017-2287-z 29411152

[B3] Anderson P. O. (2022). Guidelines for reporting cases of medication use during lactation. Breastfeed. Med. 17, 93–97. 10.1089/bfm.2021.0357 35073165

[B4] Anderson P. O. Sauberan J. B. (2016). Modeling drug passage into human milk. Clin. Pharmacol. Ther. 100, 42–52. 10.1002/cpt.377 27060684

[B5] Andreas N. J. Kampmann B. Mehring Le-Doare K. (2015). Human breast milk: a review on its composition and bioactivity. Early Hum. Dev. Spec. Issue Neonatal Update 91, 629–635. 10.1016/j.earlhumdev.2015.08.013 26375355

[B6] Bajcetic M. de Wildt S. N. Dalinghaus M. Breitkreutz J. Klingmann I. Lagler F. B. (2019). Orodispersible minitablets of enalapril for use in children with heart failure (LENA): rationale and protocol for a multicentre pharmacokinetic bridging study and follow-up safety study. Contemp. Clin. Trials Commun. 15, 100393. 10.1016/j.conctc.2019.100393 31249901 PMC6586986

[B7] Beardmore K. S. Morris J. M. Gallery E. D. M. (2002). Excretion of antihypertensive medication into human breast milk: a systematic review. Hypertens. Pregnancy 21, 85–95. 10.1081/PRG-120002912 12044345

[B8] Begg E. J. Robson R. A. Gardiner S. J. Hudson L. J. Reece P. A. Olson S. C. (2001). Quinapril and its metabolite quinaprilat in human milk. Br. J. Clin. Pharmacol. 51, 478–481. 10.1046/j.1365-2125.2001.01327.x 11422007 PMC2014479

[B9] Boberg M. Vrana M. Mehrotra A. Pearce R. E. Gaedigk A. Bhatt D. K. (2017). Age-dependent absolute abundance of hepatic carboxylesterases (CES1 and CES2) by LC-MS/MS proteomics: application to PBPK modeling of oseltamivir *in vivo* pharmacokinetics in infants. Drug Metab. Dispos. 45, 216–223. 10.1124/dmd.116.072652 27895113 PMC5267516

[B10] Bullo M. Tschumi S. Bucher B. S. Bianchetti M. G. Simonetti G. D. (2012). Pregnancy outcome following exposure to angiotensin-converting enzyme inhibitors or angiotensin receptor antagonists. Hypertension 60, 444–450. 10.1161/HYPERTENSIONAHA.112.196352 22753220

[B11] CDC (2022). Breastfeeding report card United States. Centers for disease control and prevention, national center for chronic disease prevention and health promotion, division of nutrition, physical activity and obesity. United States.

[B12] Ceulemans M. Van Calsteren K. Allegaert K. Foulon V. (2022). Information needs and counseling preferences among potential users of the future teratology information service in Belgium: a cross-sectional study involving the public and healthcare professionals. Int. J. Environ. Res. Public. Health 19, 8605. 10.3390/ijerph19148605 35886455 PMC9319400

[B13] Chowdhury R. Sinha B. Sankar M. J. Taneja S. Bhandari N. Rollins N. (2015). Breastfeeding and maternal health outcomes: a systematic review and meta-analysis. Acta Paediatr. 104, 96–113. 10.1111/apa.13102 26172878 PMC4670483

[B14] Chugh J. Dai J. Datta P. Krutsch K. (2025). Investigating the transfer of lisinopril into human milk: a quantitative analysis. J. Cardiovasc. Pharmacol. 85, 84–87. 10.1097/FJC.0000000000001642 39405564

[B15] Cloostermans L. Allegaert K. Smits A. Van Neste M. (2024). The deuterium oxide dilution method to quantify human milk intake volume of infants: a systematic Review-A contribution from the ConcePTION project. Nutrients 16, 4205. 10.3390/nu16234205 39683598 PMC11644218

[B16] Colaceci S. Giusti A. Chapin E. M. Notarangelo M. De Angelis A. Vellone E. (2015). The difficulties in antihypertensive drug prescription during lactation: is the information consistent? Breastfeed. Med 10, 468–473. 10.1089/bfm.2015.0086 26565668 PMC4683560

[B17] Davies R. O. Gomez H. J. Irvin J. D. Walker J. F. (1984). An overview of the clinical pharmacology of enalapril. Br. J. Clin. Pharmacol. 18 (Suppl. 2), 215S–229S. 10.1111/j.1365-2125.1984.tb02601.x 6099737 PMC1463484

[B18] Davis E. C. Castagna V. P. Sela D. A. Hillard M. A. Lindberg S. Mantis N. J. (2022). Gut microbiome and breast-feeding: implications for early immune development. J. Allergy Clin. Immunol. 150, 523–534. 10.1016/j.jaci.2022.07.014 36075638 PMC9463492

[B19] Devlin R. G. Fleiss P. M. (1981). Captopril in human blood and breast milk. J. Clin. Pharmacol. 21, 110–113. 10.1002/j.1552-4604.1981.tb01759.x 7014657

[B20] Duijts L. Jaddoe V. W. V. Hofman A. Moll H. A. (2010). Prolonged and exclusive breastfeeding reduces the risk of infectious diseases in infancy. Pediatrics 126, e18–e25. 10.1542/peds.2008-3256 20566605

[B21] Elung-Jensen T. Heisterberg J. Kamper A.-L. Sonne J. Strandgaard S. (2003). Blood pressure response to conventional and low-dose enalapril in chronic renal failure. Br. J. Clin. Pharmacol. 55, 139–146. 10.1046/j.1365-2125.2003.01764.x 12580985 PMC1894732

[B22] EMA (2023). Aqumeldi (enalapril). Amsterdam, Netherlands: European Medicines Agency.

[B23] EMA (2025). Aqumeldi? INN-Enalapril maleate, annex I: summary of product characteristics. Amsterdam, Netherlands: European Medicines Agency.

[B24] European Bioinformatics Institute. (1985). Compound: ENALAPRIL (CHEMBL578) Available online at: https://www.ebi.ac.uk/explore/compound/CHEMBL578.

[B25] Faisal M. Cawello W. Laeer S. The LENA Consortium (2021). Clinical pharmacokinetics of enalapril and enalaprilat in pediatric patients—A systematic review. Front. Pediatr. 9, 611322. 10.3389/fped.2021.611322 33643971 PMC7907604

[B26] Faruqi A. Patel P. Jain A. (2025). “Enalapril,” in StatPearls (Treasure Island (FL): StatPearls Publishing). 32491640

[B27] FDA (1985). Tablets: Vasotec (enalapril maleate). Washington, DC: Food Drug Administration.

[B28] Gagnier J. J. Kienle G. Altman D. G. Moher D. Sox H. Riley D. (2013). The CARE guidelines: consensus-Based clinical Case Reporting Guideline development. Glob. Adv. Health Med. 2, 38–43. 10.7453/gahmj.2013.008 24416692 PMC3833570

[B29] Gardner H. Kent J. C. Lai C. T. Mitoulas L. R. Cregan M. D. Hartmann P. E. (2015). Milk ejection patterns: an intra-individual comparison of breastfeeding and pumping | BMC Pregnancy and Childbirth | full text. BMC Pregnancy Childbirth 15, 156. 10.1186/s12884-015-0583-3 26223256 PMC4520208

[B30] Ghelfi A. M. Ferretti M. V. Staffieri G. J. (2021). Pharmacological treatment of non-severe hypertension during pregnancy, postpartum and breastfeeding. Hipertens. Riesgo Vasc. 38, 133–147. 10.1016/j.hipert.2021.01.002 33632659

[B31] Hansson M. Björkgren I. Svedenkrans J. Backman H. Hellman J. Englund-Ögge L. (2025). Setting up mother–infant pair lactation studies with biobanking for research according to regulatory requirements. Br. J. Clin. Pharmacol., 1–6. 10.1002/bcp.70201 40817578

[B32] Hurtado J. Moffett B. S. (2007). Pediatric oral formulations: a continual challenge. Int. J. Pharm. Compd. 11, 17–19. 23974478

[B33] Huttunen K. Grönhagen-Riska C. Fyhrquist F. (1989). Enalapril treatment of a nursing mother with slightly impaired renal function. Clin. Nephrol. 31, 278. 2544326

[B34] Jølving L. R. Nielsen J. Kesmodel U. S. Nielsen R. G. Beck-Nielsen S. S. Nørgård B. M. (2016). Prevalence of maternal chronic diseases during pregnancy – a nationwide population based study from 1989 to 2013. Acta Obstet. Gynecol. Scand. 95, 1295–1304. 10.1111/aogs.13007 27560844

[B35] Kaiser G. Ackerman R. Dieterle W. (1989). Benazepril and benazeprilat in human plasma and breast milk: abstracts of the IV World conference on. Clin. Pharmacol. and Ther. Eur. J. Clin. Pharmacol. 36, A1–A341. 10.1007/BF02411402

[B36] Kanic Z. Kanic V. Hojnik T. (2021). Enalapril and acute kidney injury in a hypertensive premature newborn – should it be used or not? J. Pediatr. Pharmacol. Ther. JPPT 26, 638–642. 10.5863/1551-6776-26.6.638 34421415 PMC8372859

[B37] Kappel D. Sahin L. Yao L. Thor S. Kweder S. (2023). A comparison of FDA and EMA pregnancy and lactation labeling. Clin. Pharmacol. Ther. 113, 1251–1257. 10.1002/cpt.2843 36645246

[B38] Kearns G. L. Abdel-Rahman S. M. Alander S. W. Blowey D. L. Leeder J. S. Kauffman R. E. (2003). Developmental pharmacology — drug disposition, action, and therapy in infants and children. N. Engl. J. Med. 349, 1157–1167. 10.1056/NEJMra035092 13679531

[B39] Kelly J. Doyle G. Donohue J. Laher M. Vandenburg M. Currie W. (1986). Pharmacokinetics of enalapril in normal subjects and patients with renal impairment. Br. J. Clin. Pharmacol. 21, 63–69. 10.1111/j.1365-2125.1986.tb02823.x 3004546 PMC1400811

[B40] Kent J. C. Ramsay D. T. Doherty D. Larsson M. Hartmann P. E. (2003). Response of breasts to different stimulation patterns of an electric breast pump. J. Hum. Lact. 19, 179–186. 10.1177/0890334403252473 12744535

[B41] Kiesel L. M. Bertsche A. Kiess W. Siekmeyer M. Bertsche T. Neininger M. P. (2023). Intensive care drug therapy and its potential adverse effects on blood pressure and heart rate in critically ill children. World J. Pediatr. 19, 902–911. 10.1007/s12519-023-00683-0 36854951 PMC10423157

[B42] Ku L. C. Zimmerman K. Benjamin D. K. Clark R. H. Hornik C. P. Smith P. B. (2017). Safety of enalapril in infants admitted to the neonatal intensive care unit. Pediatr. Cardiol. 38, 155–161. 10.1007/s00246-016-1496-2 27826711 PMC5288129

[B43] Kubo S. H. Cody R. J. (1985). Clinical pharmacokinetics of the angiotensin converting enzyme inhibitors. Clin. Pharmacokinet. 10, 377–391. 10.2165/00003088-198510050-00001 2994938

[B44] Leggett C. Lwin E. M. P. Ritchie U. Song Y. Gerber J. P. Turner S. (2020). Perindopril in breast milk and determination of breastfed infant exposure: a prospective observational study. Drug Des. devel. Ther. 14, 961–967. 10.2147/DDDT.S239704 32184565 PMC7060030

[B45] Marte F. Dersnah G. D. Cassagnol M. (2023). “Enalaprilat,” in StatPearls (Treasure Island (FL): StatPearls Publishing). 30485004

[B46] McCormack C. Brennand J. (2024). Postpartum hypertension, guideline for management (no. 322). Maternal clinical governance group; national health service, greater Glasgow and clyde.

[B47] MHRA (2009). ACE inhibitors and angiotensin II receptor antagonists: recommendations on how to use for breastfeeding. Med. Healthc. Prod. Regul. Agency - Drug Saf. Update 2, 3. Available online at: https://www.gov.uk/drug-safety-update/ace-inhibitors-and-angiotensin-ii-receptor-antagonists-recommendations-on-how-to-use-for-breastfeeding (Accessed September 8, 2025).

[B48] Monfort A. Macente J. Van Neste M. Huang M.-C. Nauwelaerts N. Abza G. B. (2025). Pragmatic and contextualized methods selection for safety assessment of infant systemic exposure through human milk: the Milk4baby decision tree approach - a contribution from the concePTION project. Front. Pharmacol. 16, 1602018. 10.3389/fphar.2025.1602018 40837399 PMC12361215

[B49] Nauwelaerts N. Macente J. Deferm N. Bonan R. H. Huang M.-C. Van Neste M. (2023). Generic workflow to predict medicine concentrations in human milk using physiologically-based pharmacokinetic (PBPK) modelling—A contribution from the ConcePTION project. Pharmaceutics 15, 1469. 10.3390/pharmaceutics15051469 37242712 PMC10223637

[B50] Parnizari P. Hladunewich M. A. Zipursky J. (2025). Safety of drugs in breastfeeding women with CKD. Kidney Int. Rep. 10, 2189–2201. 10.1016/j.ekir.2025.04.038 40677356 PMC12266210

[B51] Pilgrim R. Kwok M. May A. Chapman S. Jones M. D. (2025). The effect of medication use on breastfeeding continuation: a systematic review with narrative synthesis. Int. Breastfeed. J. 20, 59. 10.1186/s13006-025-00756-y 40760660 PMC12320353

[B52] Piotrkowicz E. Skrzypczyk P. Prejbisz A. Dobrowolski P. Gawlak M. Kosiński P. (2025). Safety and risks of antihypertensive medications during breastfeeding: a review of current guidelines. J. Clin. Med. 14, 3722. 10.3390/jcm14113722 40507483 PMC12156924

[B53] Ranadive S. A. Chen A. X. Serajuddin A. T. M. (1992). Relative lipophilicities and structural-pharmacological considerations of various angiotensin-converting enzyme (ACE) inhibitors. Pharm. Res. 9, 1480–1486. 10.1023/A:1015823315983 1475237

[B54] Redman C. W. G. Kelly J. G. Cooper W. D. (1990). The excretion of enalapril and enalaprilat in human breast milk. Eur. J. Clin. Pharmacol. 38, 99. 10.1007/BF00314815 2158450

[B55] Sachs H. C. Committee O. N. DRUGS Frattarelli D. A. C. Galinkin J. L. Green T. P. Johnson T. (2013). The transfer of drugs and therapeutics into human breast milk: an update on selected topics. Pediatrics 132, e796–e809. 10.1542/peds.2013-1985 23979084

[B56] Schilder J. Van den Anker J. (1995). Use of enalapril in neonatal hypertension. Acta Paediatr. 84, 1426–1428. 10.1111/j.1651-2227.1995.tb13581.x 8645963

[B57] Schwartz J. B. Taylor A. Abernethy D. O’Meara M. Farmer J. Young J. (1985). Pharmacokinetics and pharmacodynamics of enalapril in patients with congestive heart failure and patients with hypertension. J. Cardiovasc. Pharmacol. 7, 767–776. 10.1097/00005344-198507000-00023 2410720

[B58] Scime N. V. Patten S. B. Tough S. C. Chaput K. H. (2022). Maternal chronic disease and breastfeeding outcomes: a Canadian population-based study. J. Matern. Fetal Neonatal Med. 35, 1148–1155. 10.1080/14767058.2020.1743664 32208754

[B59] Sivasankar M. Raja I. N. Parthasarathy S. Suchitra S. (2025). The protective role of neonatal breastfeeding in renal health: a systematic review. J. Neonatal Surg. 14, 138–144. 10.52783/jns.v14.2216

[B60] Tabacova S. A. Kimmel C. A. (2001). Enalapril: pharmacokinetic/dynamic inferences for comparativedevelopmental toxicity. Reprod. Toxicol. 15, 467–478. 10.1016/S0890-6238(01)00161-7 11780954

[B61] Tain Y.-L. Lin Y.-J. Hsu C.-N. (2025). Breastfeeding and future cardiovascular, kidney, and metabolic health—A narrative review. Nutrients 17, 995. 10.3390/nu17060995 40290039 PMC11944316

[B62] Ulm E. H. (1983). Enalapril maleate (MK-421), a potent, nonsulfhydryl angiotensin-converting enzyme inhibitor: absorption, disposition, and metabolism in man. Drug Metab. Rev. 14, 99–110. 10.3109/03602538308991383 6301792

[B63] Ulm E. Hichens M. Gomez H. Till A. Hand E. Vassil T. (1982). Enalapril maleate and a lysine analogue (MK-521): disposition in man. Br. J. Clin. Pharmacol. 14, 357–362. 10.1111/j.1365-2125.1982.tb01991.x 6289858 PMC1427630

[B64] Van de Walle L. Vandenplas Y. Toelen J. Raaijmakers A. (2024). Vitamin D status in Belgian children: a regional study. Nutrients 16, 657. 10.3390/nu16050657 38474785 PMC10935432

[B65] Van Hecken A. Burckhardt B. B. Khalil F. de Hoon J. Klingmann I. Herbots M. (2020). Relative bioavailability of enalapril administered as orodispersible minitablets in healthy adults. Clin. Pharmacol. Drug Dev. 9, 203–213. 10.1002/cpdd.728 31411383

[B66] Van Neste M. Bogaerts A. Nauwelaerts N. Macente J. Smits A. Annaert P. (2023). Challenges related to acquisition of physiological data for physiologically based pharmacokinetic (PBPK) models in postpartum, lactating women and breastfed Infants-A contribution from the ConcePTION project. Pharmaceutics 15, 2618. 10.3390/pharmaceutics15112618 38004596 PMC10674226

[B67] Van Neste M. Nauwelaerts N. Ceulemans M. Van Calsteren K. Eerdekens A. Annaert P. (2024). Determining the exposure of maternal medicines through breastfeeding: the UmbrelLACT study Protocol—a contribution from the ConcePTION project. BMJ Paediatr. Open 8, e002385. 10.1136/bmjpo-2023-002385 38599799 PMC11015172

[B68] World Health Organization (2023). Infant and young child feeding. Available online at: https://www.who.int/news-room/fact-sheets/detail/infant-and-young-child-feeding (Accessed September 8, 2025).

