# Supplementary material for: Very low enalapril and enalaprilat exposure via human milk: a case report from the ConcePTION project
Source: Front Pharmacol. 2026 Jan 20;16:1727499. doi: 10.3389/fphar.2025.1727499 (PMC12864425; doi:10.3389/fphar.2025.1727499)
Supplement: Supplementary file 1 [file DataSheet2.pdf]

Supplementary Material

1. Bio-analysis method

Enalapril and enalaprilat concentrations were determined in human plasma and breast milk samples after protein precipitation, using reversed-phase ultra-high performance liquid chromatography (RP-UHPLC) coupled with tandem mass spectrometry (MS-MS).

The protein precipitation was performed as follows: 50 µL of sample was diluted with 50 µL blank matrix to create a common matrix (plasma/milk (1:1, v/v)). Subsequently, 600 µL methanol containing 5nM daidzein (internal standard) was added. Samples were vortexed and centrifuged for 10 minutes at 12,000g at 4°C. A 600 µL supernatant was transferred to a clean test tube and evaporated to dryness under a gentle stream of nitrogen. The dried residue was reconstituted in 200 µL water, and 5 µL was injected into the liquid chromatography tandem mass spectrometry (LCMS-MS) system.

In brief, separation was performed using a Kinetex F5 column (1.7 µm, 2.1 x 50 mm; Phenomenex, Utrecht, The Netherlands) held at 40°C. The mobile phases consisted of 0.1% formic acid in water (solvent A) and 0.1% formic acid in acetonitrile (solvent B), delivered at a flow rate of 0.4 mL/min. Gradient elution was performed as follows: 5% of solvent B during 0.5 min, increase of solvent B to 90% in 1 min which was held for 2 min followed by a decrease to 5%. The column was re-equilibrated for 3 min resulting in a total runtime of 7 min. Enalaprilat eluted at 2.80 min, enalapril at 3.05 min and the internal standard daidzein eluted at 3.00 min.

MS-MS detection was performed on a Shimadzu LCMS-8050 equipped with an electrospray ionization (ESI) source operated in positive ionization mode. The following mass transitions for the detection of the different compounds were monitored: enalapril m/z 377.20 → 234.15 (collision energy -19V), enalaprilat m/z 349.30 → 206.10 (collision energy -19V), daidzein m/z 255.25 → 181.0 (collision energy -30V).

Calibration curves were freshly prepared on the day of the analysis by serial dilution in plasma or breast milk, covering a range of 0.1 – 200 ng/mL. A $1/x^2$-weighted linear regression model was applied to adequately describe the concentration–peak area relationship. The calibration standards and quality control samples met the acceptance criteria according to the bioanalytical guideline ICH M10. Data acquisition and processing were performed using LabSolutions software.
